# Barriers and facilitators to viral hepatitis testing in Uzbekistan: scoping qualitative study among key stakeholders, healthcare workers, and the general population

**DOI:** 10.1186/s12889-024-18953-5

**Published:** 2024-06-03

**Authors:** Alyona Mazhnaya, Brogan Geurts, Krestina Brigida, Shokhista Bakieva, Shakhlo Sadirova, Annika Witzigmann, Erkin Musabaev, Michael Brandl, Heide Weishaar, Sandra Dudareva, Charbel El Bcheraoui

**Affiliations:** 1 Centre for International Health Protection, Robert Koch Institute, Berlin, Germany; 2 National University of Kyiv-Mohyla Academy, Kyiv, Ukraine; 3 Research Institute of Virology, Tashkent, Uzbekistan; 4 Department of Infectious Disease Epidemiology, Robert Koch Institute, Berlin, Germany

**Keywords:** Hepatitis testing, Central Asia, Elimination, Barriers and facilitators, Behavior change

## Abstract

**Introduction:**

In the World Health Organization European Region, an estimated 14 million people live with a chronic hepatitis B virus infection (HBV), and 12 million are affected by a hepatitis C virus infection (HCV). Uzbekistan bears a major burden of HBV and has one of the highest HCV prevalence in the region. Following a presidential decree in May 2022, significant funds were allocated to the viral hepatitis (VH) elimination program in Uzbekistan. The program expands VH testing to reach 500,000 people annually during 2022–2025 as part of the VH elimination strategy that includes the provision of free testing and affordable treatment. Exploring the existing barriers and facilitators to VH testing is pivotal for informing these interventions.

**Methods:**

This study uses a cross-sectional qualitative design to identify and explore the barriers and facilitators to VH testing among the general population in Uzbekistan. We collected data during October-November 2022 through semi-structured interviews with 12 key informants (KIs) and 7 focus group discussions with two target populations: the general population and healthcare workers (HCW) in Tashkent, Uzbekistan.

**Results:**

Following the capability-opportunity-motivation-behavior model (COM-B model) as a framework for the analysis, we identified major capability barriers to VH testing primarily linked to low health literacy and limited knowledge about VH types, symptoms, transmission, testing and treatment. Physical opportunity barriers included the time and financial costs associated with testing, diagnostics, and treatment. Sociocultural opportunity barriers involved anticipated negative reactions and stigmatization, particularly affecting women. Motivational barriers included a reluctance to be tested when asymptomatic and a general fear of receiving positive test results. The involvement of healthcare workers in promoting VH awareness and motivating the general population emerged as a facilitator.

**Conclusions:**

A multi-pronged approach is recommended to achieve VH testing goals among the general population, focusing on raising awareness and health literacy and creating an enabling environment that ensures easy accessibility and minimizing VH testing-associated costs.

**Supplementary Information:**

The online version contains supplementary material available at 10.1186/s12889-024-18953-5.

## Introduction

Viral hepatitis (VH) is a major threat to global public health. In particular, viral hepatitis B infection (HBV) and viral hepatitis C infection (HCV) can lead to chronic conditions and are the most common cause of liver cirrhosis, liver cancer and VH-related deaths [1–4]. An estimated 14 million people in the WHO European Region live with chronic HBV [3], and an estimated 12 million are affected by HCV [2]. The major burden of HBV and HCV in the European Region exists in the countries of Eastern Europe and Central Asia [1–5], with Uzbekistan having one of the highest prevalence estimates for HCV in the World Health Organization (WHO) European region [6]. A systematic review and meta-analysis of HCV prevalence in Central Asia [6] estimated a 9.6% HCV prevalence in Uzbekistan. This prevalence is lower than the pooled mean prevalence of 13.5% across Central Asia but higher than the prevalence estimates reported for each country in the region [6]. An estimated 2.5 million people were living with HBV and 1.3 million with HCV in Uzbekistan in 2016 [5]. A recent large-scale testing program estimated HBV and HCV prevalence at 3.2% and 3.0% among people attending polyclinics in Tashkent [5]. However, there is a gap in evidence for reliably estimating the prevalence of the diverse VH transmission routes in Uzbekistan [6]. Transmission routes are likely to reflect both global patterns, including perinatal transmission, unsafe injection practices, and the transfusion of unscreened blood and blood products and are further defined by country-specific context [7].

VH infections contracted in early life carry the highest risk of becoming chronic, underscoring the critical need for timely vaccination with both birth and third doses of the HBV vaccine. Uzbekistan has effectively implemented HBV vaccination policies, achieving 95% coverage for the third dose (HepB3) and 97% coverage for timely birth doses by 2020, meeting national and WHO regional targets [5, 8]. Among age cohorts born before the introduction of universal childhood HBV vaccination, the prevalence of HBV infections remains considerable, underscoring the importance of timely testing to facilitate necessary care [7]. Despite progress in HBV control, HCV remains problematic due to limited screening and high treatment costs. Efforts to integrate HCV screening with broader health services are underway [5]. These efforts are informed by the premise that in Uzbekistan, the general and key populations are affected by VH, and many infections may be attributable to nosocomial transmission [7]. 

In 2016, the WHO introduced the Global Health Sector Strategy on Global Hepatitis, aiming to make substantial progress in VH elimination worldwide by 2030 [9]. Subsequently, WHO’s Action plan for the health sector’s response to VH advocates certain country targets for HBV and HCV testing, which need to be met to reach the 2030 VH elimination goal [10]. Countries in the WHO European Region, including Uzbekistan, committed to developing corresponding national strategies and action plans supporting VH elimination [10]. Following a one-year pilot program on VH screening and treatment in primary care in Tashkent, Uzbekistan [5], which started in 2019, the Uzbek government adopted a policy which provides patients with access to free VH testing and affordable treatment [11]. The pilot project demonstrated the feasibility of simplified testing and treatment protocols for HBV and HCV in the general population in participating clinics in Tashkent [5]. During the pilot, participants were recruited among patients seeking service at the primary care level [5]; however, the pilot also identified low levels of VH awareness and knowledge among the general population. To achieve the targeted elimination goals of general population testing 500,000 people annually from 2022 to 2025, it is essential to extend outreach and testing beyond the conventional healthcare settings to include communities and groups that may not routinely access such services [5, 11, 12]. Therefore, case finding through screening, as the first critical step in the treatment and care cascade, needs to be scaled up significantly. This step is necessary to link patients to diagnosis and treatment, to prevent transmission and sequela, and, ultimately, to achieve WHO targets for HBV and HCV elimination. This requires understanding the current barriers and facilitators to VH testing in Uzbekistan to inform the development of targeted interventions and policies [5, 13]. Currently, no literature exists on this topic in Uzbekistan.

A well-defined theoretical framework is needed to systematically scope and explore the barriers and facilitators to any health behaviour. We choose the capability-opportunity-motivation-behaviour (COM-B) for its comprehensiveness and practical applicability in developing interventions [13–15]. This model helps identify interlinked factors of capability, physical and social opportunity, and motivation as influencing behaviours of providers and users of VH testing. Further, the COM-B model offers a holistic approach to exploring hindrances and enablers to targeted health behaviour [14, 15]. Its relative simplicity makes it particularly useful for creating actionable strategies in public health settings. The COM-B model draws on multiple different frameworks of behaviour change and has proven utility across various health topics such as vaccination [16–18], testing and screening [19, 20], including HCV testing [21], and adherence to treatment [22, 23].

This study aims to explore barriers and facilitators to VH testing in Uzbekistan using the perspectives of (i) key informants (KIs) as the providers of services, (ii) the general population as potential users, and (iii) healthcare workers (HCWs) who both provide and use services [13–15].

## Methods

This study follows a cross-sectional qualitative approach. Data were collected through semi-structured interviews with KIs and focus-group discussions (FGDs) with members of the general population and HCWs in Tashkent, Uzbekistan. KIs are conceptually on the supply side of VH testing; FGDs with general population participants represent the demand for VH testing in Uzbekistan, and FGDs with HCWs represent both the demand and supply sides.

Data collection was designed to identify the interlinked individual (capability, motivation) and context (physical and sociocultural opportunity) determinants of health behaviours and was guided by the COM-B model [14, 15]. Briefly, capability covers mental and physical ability and the capability of people to provide or get tested for VH. It includes several concepts such as knowledge, skills, and physical ability [14, 15]. Motivation relates to automatic and reflective factors that affect individuals’ readiness or willingness to engage in VH testing [14, 15]. Physical opportunity includes the structural, legislative, and health-system-related context for VH testing [14, 15]. Lastly, sociocultural opportunity elucidates the context for VH defined by cultural traditions, cues, norms, values and beliefs, social demands, and social support [14, 15]. We have chosen to use “sociocultural opportunity” to better capture the impact of cultural influences, particularly relevant in Uzbekistan. This choice aligns with the recent European framework emphasizing cultural considerations in health policies and services [24]. We chose key informant interviews (KIIs) to involve stakeholders with diverse roles and positions within the Uzbekistan VH program, ensuring privacy and gathering diverse perspectives from key figures closely involved in various aspects of VH testing. The main goal of employing FGDs was to explore participants’ perceptions, opinions, beliefs, and attitudes towards VH testing, providing a collective environment that enriches the data through dynamic group interactions and enables examination of how people think and feel about the issue.

### Participant selection and recruitment

First, potential KIs were identified through a desk review of the VH program in Uzbekistan and scoping interviews conducted in Tashkent with country-level experts. KIs were then sampled and recruited from different levels and settings of the supply side of VH testing, including policymakers, specialist doctors and nurses, and health facility managers or administrators. This included sampling representatives of different institutions involved in VH testing policy, guidelines, implementation, and monitoring. Key informant topic guides (Supplementary file 1) were organized around five areas: organization background and context, challenges for VH testing, testing success and opportunities and improving VH testing in Uzbekistan.

Second, FGDs participants were recruited from the general population and among HCWs in Tashkent, Uzbekistan. Members of the general population were recruited using outreach through local community committees (*mahallas*), large governmental employers, and outreach at gathering places, such as large markets. HCWs, who were selected due to both being a specific target group for VH testing and as key individuals who communicate about VH testing with members of the general population, were recruited via a direct invitation to participate in the study from healthcare institutions across Tashkent. FGDs were organized separately for HCWs and members of the general population, and with homogeneous compositions based on the disaggregated demographics: gender, education, and employment status (i.e. higher educated male members of the general population, lower educated male members of the general population, employed female members of the general population, unemployed female members of the general population, male HCWs, two FG with female HCWs). All participants, with the exception of few HCWs, were recruited from various locations across Tashkent without any prior contact or engagement between the participants.

The topic guide for FGDs focused on the participants’ experiences and perceptions of barriers and facilitators of VH testing, was organized following the COM-B model, and covered the following topics: general health seeking-behaviour, knowledge about VH testing (capability), access to VH testing (physical opportunity), views on VH testing (motivation), other people’s influences on access (sociocultural opportunity), and ideas for improving VH testing in Uzbekistan. The topic guide was refined based on several scoping interviews and field visits to testing locations in October 2022. The research and data collection team piloted the topic guide before data collection.

The Ministry of Health of the Republic of Uzbekistan Ethical Committee reviewed and approved the study on November 4th, 2022 (minutes of the meeting #7/2-1706).

### Data collection

Data were collected between October and November 2022. All KIs and FGDs participants provided written and verbal informed consent before the interview or focus group discussion began. All participants were asked for their consent to be quoted in publications and had the option to consent to participate in the study without being directly quoted. KIs were also asked to voluntarily provide their profession, organizational affiliation, and basic demographic data (age group, gender, and years of professional experience). Focus group participants were similarly asked to voluntarily provide their socidemographics (age, gender, education level, and profession). Participants received compensation for travel and time, equivalent to ~ 13 USD each. All KIIs and FGDs were conducted by a local data collection team in Tashkent in either Uzbek or Russian. Notes were taken, and all KIIs and FGDs were audio recorded, transcribed verbatim and translated into English by a professional company. All directly identifying information, such as names and specific locations, were removed before analysis.

### Data analysis

The analysis was guided by the study’s conceptual framework (COM-B model) and aimed at identifying barriers and facilitators of VH testing. Analysis was performed following a rapid assessment procedure (RAP) approach [25, 26] by AM, BG and AW. The RAP approach organizes and summarizes the data according to a pre-defined framework [27]. Separate RAP sheets for KIIs and FGDs were developed based on the topic guide and the COM-B model. Each RAP sheet was piloted and amended using one transcript. Each key informant transcript was then summarized by one researcher in the team and verified by another researcher in English or Russian. Attention was given to areas of agreement among participants as well as areas of discordance. FGDs transcripts were summarized separately with a RAP sheet designated for each type of groups (e.g. men, women, and HCWs). The research team then sequentially summarised and structured the factors according to COM-B factors. All RAP sheets and summaries were compared to identify areas of saturation, pertinent barriers and facilitators, and areas of discordance. The interrelatedness of different barriers and facilitators and specific recommendations made during KIIs and FGDs were further developed to suggest areas for further in-depth investigation and potential areas of intervention to scale up and improve VH testing.

## Results

### Participants

A total of 12 face-to-face KIIs were conducted and lasted on average 30 min (min = 12, max = 45). KIs represent various levels of Uzbekistan’s health system, from direct service provision to participation in developing national VH policies. Six KIs had more than 20 years of professional experience; three KIs had less than ten years of experience (median = 19.5, min = 3, max = 42, IQR 7–30).

Seven FGDs were conducted with 44 individuals, lasting on average 90 min (min = 76, max = 106). In total, 32 FGD participants were female, and 12 were male; their ages varied between 24 and 60 years, with a median of 42.5 years (IQR 34–45). The FGDs were comprised of unemployed female homemakers (*n* = 6), females employed outside the health sector (*n* = 6), employed males with higher levels of education (*n* = 6), and employed males with lower levels of education (*n* = 6), and HCWs at different levels of service provision (*n* = 18). A summary of key informants and focus group participants’ characteristics can be found in Table 1.

**Table 1:** Summary of KII and FGD Participants Characteristics

Participants’ characteristics	Female	Male	Total
**Key Informants (** ***N*** ** = 12)**	**6**	**6**	**12**
Years of professional experience, median (IQR)	14 (5–32)	22 (3–42)	20 (3–42)
**Focus Groups (FG) (** ***N*** ** = 7)**	**32**	**12**	**44**
Age, median (IQR)	42 (24–66)	43 (26–44)	43 (22–66)
Composition of FG			
Healthcare workers	18		18
Unemployed	6		6
Employed, lower education		6	
Employed, higher education	8	6	14
Employment status			
Employed	26	12	38
Unemployed	6	0	6
Education level			
Secondary education	3	3	6
Professional education	9	3	12
Higher education	20	6	26

### Views on VH testing in Uzbekistan

KIs and FGDs participants’ views on VH testing in Uzbekistan and perceived barriers and facilitators to conducting and scaling up VH testing in Uzbekistan are summarized according to the COM-B factors [11, 15] (Table 2). We also report any differences in the barriers and facilitators described by different types of participants.

**Table 2:** List of identified barriers and facilitators for VH testing in Uzbekistan categorized into capability, opportunity, and motivation factors

	Identified Barriers	Identified Facilitators
Capability	Low health literacy (general and VH-specific), limiting informed decision-making about health and VH. Insufficient understanding of the importance of screening in the absence of symptoms. Predominant perception of VH as a childhood disease or misunderstanding of its various types. Limited awareness regarding the locations and procedures for VH testing.	Improved knowledge and understanding of VH among participants with higher education levels. Enhanced awareness and understanding of VH among healthcare workers. Awareness about free testing, the importance of screening, risk during dental/surgical procedures, and mandatory testing for specific groups.
Opportunity		
Physical	High or uncertain costs associated with VH tests and subsequent treatments. Time constraints and inconvenience associated with testing procedures, particularly in governmental institutions. Variability in the perceived quality and availability of tests across different healthcare institutions.	Availability of free testing and treatments. Introduction of free mandatory testing for specific professions. Funding for material procurement as part of efforts for VH elimination Pilot program offering free testing Screening women during pregnancy
Sociocultural	Anticipated negative reactions or lack of support from family and social circles, especially towards women. Lack of clear guidelines for reporting positive test results to contacts, potentially leading to familial discord. Fear of stigmatization among individuals with positive VH test results.	Support anticipated from family and social circles when discussing VH testing. Increased awareness and understanding of VH transmission routes among the population with higher levels of education.
Motivation	Low priority given to health and preventative screening in the absence of symptoms among the general population. Fear associated with potential positive results, treatment costs, and social stigma. Fear of getting infected during testing. Fear of the unknown consequences of a positive result.	Presence of symptoms or feeling unwell. Desire to protect others, especially family members and close relatives. Testing mandates for certain groups and professions. Responsibility felt by women to maintain health for family care. Healthcare workers’ professional responsibility to protect others and routine testing practices.

### Capability

#### Health literacy: general and VH-specific

Low health literacy, which encompasses the personal knowledge and competencies necessary for informed decision-making about health [28] and VH, was proposed as one of the key barriers to people’s uptake of VH testing services. Almost all participants stressed the importance of education on VH for the general population. According to patronage nurses, who conduct reach out in the community and provide home-based care to patients, people often believe that VH is incurable. KIs and FGD participants emphasized that a general lack of knowledge about VH in general and the availability of testing and treatment is a major barrier to getting tested. Some KIs suggested that health is not considered a priority in Uzbek society, contributing to a low motivation for screening and testing unless one is experiencing symptoms. According to KIs, the concept of screening is not widely understood among the public. They overwhelmingly agreed that there is a lack of knowledge among the general population about the need for VH testing without experiencing symptoms, and it was a major challenge to VH testing scale-up.

*“Then, there should be more awareness about this [VH] among the population. They are afraid that they will be infected even if they shake hands. Hepatitis A can be transmitted by this way, but hepatitis B and C are not. We need to explain this properly*.” (49-year-old female nurse).

*“I think it is due to the population’s low knowledge or medical culture and the low importance of health…Patients come to doctors’ consultations only when they are severely ill and lying down.” (Infectious disease specialist)*.

All FGDs participants reported knowing something about VH and understanding it as a serious infectious disease. However, while hepatitis A in children was most discussed, individuals with higher education levels were more inclined to discuss also other types of VH, including specific symptoms and available treatment options. HCWs participating in the FGDs knew more about prevalence among certain groups and treatment options. They also expressed confidence in their knowledge to make informed decisions for themselves and consult their patients.

*“I know that there are several types of hepatitis - these are A, B, and C, I think, and there is some other type of hepatitis. And I know that hepatitis A is transmitted through contact, through dirty hands. If one person in the family gets sick, it can go to another family member. But B and C are exclusively sexual. I know that they are successfully treated.” (45-year-old female sales representative)*.

KIs and FGDs participants suggested the most important step in increasing VH knowledge and awareness, and hence the testing behaviour, is to increase ‘*propaganda*’ (female nurse) surrounding VH testing. The proposed components of such ‘*propaganda*’ included mass-communication campaigns targeted at increasing general knowledge about VH, the availability of VH testing free of cost, the importance of VH screening independent of whether symptoms are experienced or not, the risk of getting VH while receiving dental or surgical procedures and reminding about mandatory testing for specific groups. Participants also highlighted that mass communication campaigns should be used to improve general health literacy.

*“Although free screening is being carried out, I believe there are still some obstacles for the population to come to this examination. First, there is the fact that the population does not have the required level of information about viral hepatitis B and viral hepatitis C. When they are well-educated about the disease, when they know the complications of the disease and what can happen next, they will get tested and do the necessary things to, …treat and prevent it. So, I think that’s the first obstacle.” (VH specialist)*.

#### Knowledge of VH testing: when, where, and how to get tested

Participants commonly reported the perception that a person should get tested for VH when feeling unwell, ‘having a problem’, or when somebody in their close circle tested positive. Many participants from the general population understood that testing is available and common at healthcare institutions, including at the local polyclinic or family doctor, specialized institutions, and private clinics. Perceptions about the quality of care and affordability determined which institution people preferred for VH testing. Polyclinics and state institutions were regarded as varying in quality and availability of testing materials. Polyclinics were distinguished as conducting lower quality tests compared to private or specialized state institutions. Polyclinics, however, were reported as somewhat preferred by participants with lower levels of education and employment, whereas other participants preferred private clinics. KIs, on the other hand, identified private clinics as possible risk areas for VH due to less stringent regulatory and sanitation mechanisms.

*“I do not like to get tested in these state polyclinics because I fear these conditions … There is a risk of infection. On the contrary… yes… it is better to go to a private clinic. Well…it is better to pay than to risk.” (30-year-old female sales representative)*.

Some participants preferred to be tested in a specialized infectious disease hospital as they would not want to potentially be tested twice at two different locations (screening and confirmatory). Participants’ knowledge about the cost of testing varied from being free in some places to citing prices as high as approximately 500 000 Uzbekistan Sum (about 40 EUR). Participants who were unaware of the cost expected it would be high. Overall, there was no clear understanding for whom, when and where testing is free of charge.

### Opportunity

The opportunity barriers and facilitators for VH testing consisted of physical opportunity and sociocultural opportunity. Physical opportunity included considerations of cost/funding, time, and perceived quality of tests. Sociocultural opportunity included support and anticipated reaction of the family and political commitment mentioned by KIs.

#### Cost of VH and funding

The high cost of the VH test, either known or anticipated, was a key barrier mentioned by FGD participants. Only a few participants mentioned the availability of free testing, and those who were aware were not sure who was eligible to receive it. Nevertheless, if tests were free and readily available, most thought this would encourage more people to access testing. HCWs in FGDs identified cost as the major barrier to testing, particularly for those with lower incomes. For this reason, the pilot program implemented in Uzbekistan in 2019–2020 was perceived by participants as very effective in identifying new cases because it offered free testing. HCWs also welcomed the introduction of free mandatory testing for certain professions. HCWs described that some free tests were available in polyclinics at the time of data collection. However, they mentioned they were mostly used for pregnant women and were not enough in quantity to cover the overall need.

*“In 2020, we tested the population… for hepatitis B and C. And then, indeed, we identified a lot of patients with hepatitis B and C. And, thanks to this company that conducted the study, all testing was free. And there was free treatment. And, of course, many patients improved their health then. I am also in favor of free testing among the population in our Republic, in particular, in our polyclinic.” (60-year-old female physician)*.

Anticipated costs related to confirmatory testing and treatment in case of positive results were cited as an additional deterrent to getting tested for VH. From the perspective of KIs, the lack of national funding for confirmatory testing and treatment was an ongoing problem but should be addressed with scaled-up government funding in 2023.

#### Time for testing and follow-up

Aligning with available times to get tested and following up on the results was seen as another barrier. Participants described that getting tested in polyclinics or specialized medical intuitions meant carving out time during working hours, which was a particular barrier for those employed. The time needed to undergo the test was short, however, travelling to the testing place, queuing, and administrative procedures at governmental clinics often took away from the time needed for other priorities. Thus, time was another reason why some participants preferred private clinics, which were described as providing more flexibility in testing hours, having the ability to perform screening and confirmatory testing, and providing testing results quicker.

*“To make it better, I’m telling you, we have queues everywhere, our problem is queues, everywhere. This is our problem.” (Head nurse)*.

KIs hypothesized that with the introduction of free testing for the general population, time-associated costs would become the main barrier as queues would increase in state polyclinics. KIs understood that it would be challenging for those employed to explain why they leave the workplace for testing without symptoms, particularly for temporary workers, street workers, and businessmen. KIs hypothesized that the main barrier to motivating men to get tested was the requirement to disclose that they were getting tested. KIs recommended additional outreach and mobile testing efforts, including mass testing at the workplace.

#### Role of the family

When discussing VH, participants generally expected to receive support for getting tested or upon receiving positive results from their family members. Male FGD participants, in particular, did not seem to be concerned about negative reactions from their social circle towards them getting tested for VH. Many unemployed women reported that their families understood the need for testing when it was related to employment or travel requirements. However, some participants also reported that young women need to ask permission from family members to be tested, particularly if it impacts their familial duties.

Both KIs and FGD participants highlighted a notable gender-based enforcement of the requirement to undergo testing before marriage, impacting women more than men. Furthermore, participants explained that women were more likely than men to anticipate stigma from family members in the event of a positive VH test result. Whether women felt supported or concerned about stigmatization seemed related to their own level of education and that of their family members and people in their community. Some participants reflected that the opinions of others should not matter when a person is taking care of their health.

*“I believe that it also depends on the awareness of this infection. And various strata of the population who do not know about it… Yes… And if they hear that a person has hepatitis, they will move away from this person… ahh… cut off contact with him. And I believe that if more intelligent people, they will not be afraid.” (40-year-old female manager)*.

HCWs believe that daughters-in-law were most at risk of not being supported for testing or in case of positive test results. However, most HCWs suggested if people are educated about transmission routes, they would be more likely to support someone in the family to go for testing. Similarly, HCWs perceived avoiding contact with those who tested positive as a result of the lack of knowledge about types of VH and transmission routes.

*“[positive test result] will change the relationship of the surrounding people to him. People may withdraw or distance. “Will infect me,” they think. Because many people do not understand this disease well, they think that can get infected if I hold hand, eat with the person, or drink water.” (27-year-old female nurse)*.

KIs described VH as less stigmatized than other infectious diseases, especially other STIs and HIV. KIs also noted that guidelines for reporting positive test results to contacts, specifically spouses and sexual partners, were unclear. KIs described a fear that reporting positive test results to contacts might cause family issues in case of gaps in knowledge about the disease and its transmission routes.

#### Role of healthcare workers

HCWs perceived that they play a key role in motivating people to get tested by conveying information. HCWs themselves were used to yearly testing and perceived it as routine, however, some still worried every time before receiving the results, therefore they could understand patients’ concerns.

*“Well, first of all, we are doctors. Conduct counselling. Tell him what will give him this testing. How important is early detection and the benefits of early treatment of this disease.” (49-year-old female general practitioner)*.

HCWs also discussed the importance of engaging with family members once a patient tests positive.

*I also talk to our family members very often. I will also involve them to get checked. I think it is good to prevent the disease. (24-year-old female nurse)*

#### Political commitment

KIs overwhelmingly voiced appreciation, confidence, and the positive impact of the political commitment to eliminating VH through the Presidential decree and National program. Government support was perceived as the most essential and impactful factor necessary to achieve high VH testing rates. According to KIs, the high prevalence and incidence of VH in Uzbekistan has resulted in a high level of political commitment. Examples of political support were the introduction of free testing and treatment, high targets for testing and providing funding, procurement of materials, increased VH research, and overall general polyclinic funding.

### Motivation

FGD participants offered various ideas about what would motivate people to get tested. Perceived motivating factors for testing included having symptoms and feeling unwell, wanting to protect others, testing mandates, and fear.

#### Testing without symptoms

For male participants from the general population, experiencing symptoms (e.g., fatigue, nausea, pain, discomfort, bleeding or enlarged liver) were the most salient reasons to get tested. According to FGD male participants those who feel healthy do not see the reasons to get tested. HCWs also reflected on the challenge of encouraging people to test for VH when they are not experiencing symptoms.

*“Most of the time, our patients come only when there is pain and discomfort. Or if the operation is necessary, they will force themselves for testing. [They] do not come and check on own will.” (38-year-old female entrepreneur)*.

Female participants talked more about being motivated to get tested before or after encounters with the healthcare system, e.g. before surgery, during pregnancy, or when receiving dental or cosmetic services. Some female participants also mentioned their responsibility to stay healthy and care for their families as a motivating factor. HCWs reported women often learn about their VH status when testing during pregnancy.

*“.more often among pregnant women carriers of viral hepatitis are detected. As a rule, pregnant women do not know and only find out when they are tested for hepatitis in connection with pregnancy. (60-year-old female physician)*

#### Testing to protect others

Getting tested to protect others was an important facilitator, mentioned by all categories of participants. For participants from the general population, testing is perceived as a critical preventive measure that can help protect their children and other relatives. On the other hand, HCWs mention that their motivation to protect others derives from their professional responsibilities.

#### Mandatory testing

There was almost a unanimous idea among all participants that mandatory VH testing for certain groups was the most effective way to get people tested if they did not experience symptoms. KIs, for example, thought that voluntary testing for vulnerable groups such as children born to VH-positive mothers, people who are incarcerated, migrants, and individuals who are HIV-positive, decreased the ability to detect VH among people who are at the highest risk. FGD participants were aware of some testing mandates and also were supportive of them.

*I think it is a bit more difficult for us to go and get checked on our own will, this is due to our mentality. To prevent this disease, …. This is only possible if those employees [who work with VH, in kindergarten, schools] use mandatory testing rules. We often do it only when something is mandatory. (28-year-old male teacher)*

KIs also mentioned requirements to get tested before getting married as an important factor. However, communication about the need to be tested was seen as a challenge, as young people may not fully understand the risk factors. Some participants described instances where this caused issues in the family and possible stigma.

*Screening for hepatitis in the population is not so common…they are forced to get checked only when starting a job or before getting married. I don’t think the rest matters much. (35-year-old female entrepreneur)*

HCWs supported the necessity and benefits of regular testing for themselves, describing mandated testing as crucial and beneficial. Additionally, HCWs suggested that the absence of similar testing requirements for the general population was contributing to the low rates of VH testing.

*“…because we are in the risk group, once a year, we submit hepatitis B and C tests in yearly medical examination. Because we are in contact with different patients. Whether we know it or not. There may be technical breaches. I think it is very useful for our health. If the disease is detected early… for example, the medical staff checked for timely detection and treatment.” (49- year-old female nurse)*.

#### Fear

Fear of becoming infected while receiving medical services, receiving a positive test result, the subsequent costs related to diagnosis and treatment, unknown consequences of the positive test result, and a fear of family reaction were further identified as strong (de-)motivators for testing. These factors differed according to knowledge about the disease as well as social and physical circumstances among the FGD participants.

Concerning the fear of social consequences, anonymous testing was proposed as a potential method to increase the uptake of VH testing. KIs similarly suggested that the requirement to show identification for testing was a barrier but thought VH was less stigmatized than other infectious diseases and that identifying the client was important to assure continuity of diagnostics and continuity throughout the treatment cascade.

## Discussion

This is the first qualitative study in Uzbekistan to explore and map views on VH testing among key stakeholders, the general population, and HCWs. It uncovers barriers and facilitators relating to capability, opportunity and motivation for VH testing in Uzbekistan and their interrelatedness. This study further provides insights into areas that should be further studied and addressed to scale up and improve VH testing in the country. The results indicate potential challenges with scaling up VH testing in Uzbekistan and present an evidence base for developing complex multi-level interventions. Our findings further contribute to the emerging evidence on VH testing in Central Asia – a critical region for worldwide VH elimination efforts.

The analysis shows that low health literacy and gaps in knowledge about VH types, symptoms, transmission routes, and treatment options among the general population are important capability barriers to VH testing. The most prominent physical opportunity barriers are the time and financial costs associated with getting tested, with the confirmation of test results and treatment. Sociocultural opportunity barriers include anticipated negative reactions and stigmatization from family members, especially relevant for women. These factors are mediated by education and health literacy. The most salient motivational barriers were reluctance to getting tested without symptoms and a fear of a positive test result. HCWs who understand their crucial role and unique position in encouraging public testing can increase societal knowledge and awareness of VH. Combined with the substantial political commitment already noted by KIs, this approach holds significant potential to enhance knowledge and motivation related to VH within the population.

Barriers to VH testing do not act in isolation but often influence and manifest together. Various interrelated barriers deter people from getting tested for VH in Uzbekistan and prevent a substantial increase in testing uptake. To scale up VH testing effectively and sustainably in the country, it is necessary to address these barriers systematically, jointly, and holistically. While mass campaigns might increase awareness and knowledge about VH and the availability of free testing, they will not be sufficient to substantially increase VH testing unless barriers related to time, cost, anticipated and experienced stigma, and motivation are addressed. Further, meeting the elimination targets through substantial scale-up in testing and treatment requires simplifying care pathways from screening to treatment.

Stigma is one of the barriers to VH testing that illustrates the interrelatedness of factors, particularly as stigma is a multilayered and multi-type social phenomenon that affects mental and physical health [29]. Public health researchers and practitioners increasingly recognize the importance of addressing stigma for improving health outcomes. In this study, stigma surfaces as part of sociocultural opportunity barriers, specifically as anticipated or experienced stigma associated with getting tested or sharing positive test results. Stigma also becomes apparent in the capability barriers, with people having skewed perceptions of VH which lead to stigmatization and influences physical barriers such as a reluctance to disclose a visit to the health facility to get tested to their employer. The exploratory nature of this study does not allow us to fully understand the role of stigma on VH testing in Uzbekistan, which warrants further investigation. Nevertheless, universal healthcare includes non-stigmatizing and voluntary services [30], which means that any effort to increase VH testing has to consider potential unintended negative effects and aim to improve equity and acceptability [15]. Although mandates are considered effective in increasing VH testing rates, it is crucial to assess and mitigate any possible stigmatizing effects that such compulsory measures might entail. Our findings on the prominence of stigma are particularly interesting given that our study investigates barriers to VH testing among the general population and not specifically among high-risk groups, among who anticipated and experienced stigmatization can be expected to be even higher.

Lack of knowledge about VH as a barrier to VH testing is not a finding that is unique to this study but is reported in other settings and populations [30]. Interventions to address knowledge and capability barriers should be built on existing evidence. Doing so can ensure that interventions are appropriate and evidence-based and include information that avoids further stigmatization of those getting tested or receiving positive test results. For example, a recent study on increasing HCV screening reported no independent effect of HCV knowledge on receipt of testing, suggesting that risk perception, particularly birth cohort status, may be a more influential factor than knowledge alone [31]. However, the relationship between knowledge, risk perception, and testing behavior warrants further investigation in the context of Uzbekistan. Existing evidence, however, suggests that focusing communication efforts on risks and the role of VH testing in decreasing risk [32] could be a promising way forward.

Stakeholders who participated in this study confirm that increasing VH testing is a high health priority in Uzbekistan. Starting in 2023, the government of Uzbekistan committed to free screening and confirmatory testing for the general population, including setting an ambitious target to test 2 million people per year [11, 12]. Additionally, starting in 2023, patients with VH in Uzbekistan can receive free treatment under the National Program for Viral Hepatitis Elimination [11] which significantly contributes to attaining the WHO 2030 goals. This policy may overcome the aforementioned cost barriers but may also exacerbate waiting times, another key physical opportunity barrier. To overcome these barriers, environmental restructuring in the form of flexible hours for testing, an increase in the number of testing venues and staff, outreach initiatives which allow workplace or remote testing, and electronic invitation, appointment, and reminder systems should be considered given their successful application in other settings [15, 30, 31, 33, 34]. Planning authorities should consider the cost of tests, the associated human resources, and operational expenses. To achieve the elimination targets, it is essential to simplify care pathways accordingly and ensure that the health system is equipped with the necessary resources to support and sustain these efforts effectively. Further, our results suggest that addressing the lack of trust in governmental healthcare settings is imperative, as participants expressed concerns regarding the quality of VH tests conducted in these facilities and feared potential risks of infection due to perceived suboptimal conditions.

Given the critical role of HCWs in offering and conducting VH testing, their empowerment and engagement in scaling up VH testing should be a further priority on the VH elimination agenda in Uzbekistan. Other studies suggest that HCWs’ knowledge, confidence in the quality of tests, awareness of existing policies, and perceptions of social endorsement and support from colleagues can increase VH testing [15, 30, 33].

Finally, it is important to reflect on the limitations of this study. First, this is a qualitative study with rapid data collection and analysis aimed at mapping the most salient barriers and facilitators to VH testing in Uzbekistan. The insights gained can be useful for documenting, systematizing, and informing policy, interventions and communication while scaling up VH testing. Nevertheless, the data do not represent the entire population of Uzbekistan as the data was collected in Tashkent and among specific groups only. Confirmation at a larger scale would be useful. We conducted targeted sampling by different demographic strata. However, people more open to sharing their experiences and with no other pressing critical concerns may have been more likely to decide to participate in this study. The experience of representatives of key populations and particularly high-risk groups would greatly enrich the understanding of barriers and facilitators to VH testing in Uzbekistan. Second, due to the exploratory nature of this study, the recruitment of KIs and FGD participants did not follow a saturation concept, but a predefined number based on the researchers’ experience and cross-sectional timeframe. However, we were able to identify recurrent themes, suggesting that saturation was reached within the study sample within the analysis phase. Third, time and resources did not allow us to interview those with recent experience obtaining VH testing in Uzbekistan, which would be valuable to understanding lived experiences of those accessing existing services. Future studies could address these limitations. Further, we acknowledge the necessity of a deeper mixed-methods examination of the barriers and facilitators of VH testing identified in this study to understand their variability, nuances, and implications thoroughly. We further suggest that quantitative studies are conducted to identify variables which are associated with VH testing. The limitations of our study highlight the importance of adopting a comprehensive approach in future research phases, ensuring a robust exploration of these factors within the context of VH testing in Uzbekistan.

## Conclusion

This study describes salient interlinked barriers and facilitators to VH testing as perceived by the general population, HCWs, and key stakeholders in Uzbekistan. To reach VH testing goals, a multi-pronged approach, including raising awareness and health literacy of the population and creating an enabling environment that makes VH testing easily accessible with little to no associated cost for the patient is key. The involvement of HCWs in promoting awareness, providing guidance, and delivering testing services can significantly enhance testing uptake. Therefore, alongside efforts to raise awareness and improve accessibility, engaging and empowering HCWs should be prioritized to strengthen the effectiveness of VH testing initiatives. For this approach to be effective actions to increase and maintain trust in the healthcare system through governmental leadership are required.

### Electronic supplementary material

Below is the link to the electronic supplementary material.

Supplementary Material 1

Supplementary Material 2

## Data Availability

The datasets generated and analyzed during the current study are not publicly available for sensitivity reasons but summary sheets are available from the last author on reasonable request.
