# Supplementary material for: Barriers and facilitators to viral hepatitis testing in Uzbekistan: scoping qualitative study among key stakeholders, healthcare workers, and the general population
Source: BMC Public Health. 2024 Jun 3;24:1482. doi: 10.1186/s12889-024-18953-5 (PMC11145832; doi:10.1186/s12889-024-18953-5)
Supplement: Supplementary file 1 — Supplementary Material 1 [file 12889_2024_18953_MOESM1_ESM.docx]

**Focus Group Discussion Topic Guide
(general public, non-pregnant mothers of young children, tattoo artists, medical staff, or other higher risk groups, etc.)**

**Barriers and Facilitators to Viral Hepatitis Testing in Uzbekistan – COVIMPACT**

**Exploratory research objectives:**

1. Identify key barriers and facilitators to VH testing in Uzbekistan.
2. Examine different perspectives on, and mechanisms behind, the key barriers and facilitators to VH testing in Uzbekistan.

*Italics indicate instructions for the interviewer and should not be read out.*

| ***Welcome and introduction to the study***   - *Thank the participants for agreeing to participate in the group discussion.* - *Introduce the facilitator(s).* - *Give a brief introduction to the study, including:  - topic,  - collaborating partners,  - selection of participants,  - data collection method,  - ethics and data protection (including data sharing, confidentiality, voluntary participation), - incentives.* - *Obtain socio-demographic data.* - *Obtain consent and start recording.* |
| --- |
| ***Rules for the focus group***   - *We ask you not to disclose what has been said and who said it outside the group.* - *Please be respectful of others. One person speaking at a time.* - *Please say your number each time before you speak.* - *Please make sure your phones are on silent.* |
| **Probing:**   - If someone discloses their hepatitis status or that they have tested for it before, acknowledge it in subsequent probes.  If they haven’t disclosed this information, keep the probes general and refer to “someone who might want to get tested”. - Does anyone have something else to add? - Does anyone else have a different opinion or experience? Then **pause long enough to allow reflection and responses.** |

|  | **Topic** | **Main Questions** | **Probes** |
| --- | --- | --- | --- |
|  | Introduction | **Can you start by briefly introducing yourself and why you have decided to participate today?** | |
| 1 | Health services | **Can you briefly tell me how you normally seek help if you are not feeling well or have a health-related concern? This does not have to involve going to a doctor or health center.** | Why do you do this/Why do you take this route?  Do you speak with family or friends about your concern?  What types of services do you normally look for/use?  What is most important when you seek help for your health? |
| 2 | Knowledge about VH testing (capability)  Access to VH testing (physical opportunity) | **Today we are talking about hepatitis and hepatitis testing in Uzbekistan. What do you know about hepatitis?**  **What can you do if you think you have hepatitis? Can you walk us through the process of what you could do?**  **What else do you know about hepatitis testing in Uzbekistan?** | Who/Which groups are most affected by hepatitis?  Are there other words people use for hepatitis?  How do you know what you know about hepatitis?  Do you feel you know enough about hepatitis?  *Probes on VH services:*  Where do you go to receive VH testing? Is there a place you prefer?  Can you be tested close to where you live?  Who can access the service?  How much does the service cost?  How long would it normally take to do the test?  How long before you get the results? |
|  | *[If not already identified]* **There are different types of hepatitis which can affect any person. Tests to see if someone has hepatitis are usually done by taking a blood sample.** | | |
| 3 | Views on VH testing (motivation) | **What do you think are the reasons someone might want to get tested for hepatitis?**  **Can you describe the challenges someone might face when trying to get tested for hepatitis?**  **Do you think it is important to be tested for hepatitis? Why or why not?** | Is it easy to get tested for hepatitis?  *Probe: physical barriers, time, location, environment, people’s opinions* |
| 4 | Other people influence access (social opportunity) | **How comfortable would you feel asking (a healthcare worker) to be tested for hepatitis?**  **Have you ever discussed being tested for hepatitis with anyone? Your colleagues, friends, family? Why?** | Is there anyone who has encouraged you to be tested? *Probe: Health care worker, religious leader, colleague, friend*  How would your friends, family, or colleagues react if they knew you were going for a hepatitis test?  Would this change if you were testing for different types of hepatitis?  How do you think you would react if you tested positive?  How would colleagues, friends or family react if they knew you tested positive? |
| 5 | Ideas for improving VH testing | **What do you think would have to change so more people get tested for hepatitis in Uzbekistan?** | Do you think different things need to happen in order to increase testing in Tashkent compared to outside the capital?  Who should make these changes? |
| 7 | Closing | **Do you have anything you want to add about hepatitis testing that we have not covered?**  *Thank participants. Remind them they can contact you if they have any questions. Remind them of the rules about confidentiality.* | |
