# Supplementary material for: Barriers and facilitators to viral hepatitis testing in Uzbekistan: scoping qualitative study among key stakeholders, healthcare workers, and the general population
Source: BMC Public Health. 2024 Jun 3;24:1482. doi: 10.1186/s12889-024-18953-5 (PMC11145832; doi:10.1186/s12889-024-18953-5)
Supplement: Supplementary file 2 — Supplementary Material 2 [file 12889_2024_18953_MOESM2_ESM.docx]

**Mapping Barriers and Facilitators to Viral hepatitis Testing in Uzbekistan – COVIMPACT**

**Key Informant Topic Guide
(Policy makers, hospital representatives, advocacy groups, etc)**

*Italics indicate instructions for the interviewer and should not be read out.*

1. **Introduction and Consent**

|  | **Main Questions** | **Probes** |
| --- | --- | --- |
| 1.0 | ***Welcome and Introduction to the Study***  *Thank the key informant for agreeing to participate in the interview.*  *Introduce the interviewer(s).*  *Give a brief introduction to the study, including:  - topic,  - collaborating partners,  - selection of participants,  - data collection method,  - ethics and data protection (data sharing, confidentiality, voluntary participation), - incentives.* | |
| 1.1 | ***Consent***  Have you read the consent form? Do you have any questions?  Do you give us your consent to participate in this research? | *Start the audio recording*  *Ensure to record the consent. The person should state their name and that they have received the information sheet, understood everything and have no further questions, and consent to participate in the study.* |
| 1.1.2 | ***Demographic information & introduction***  **To begin I would like to ask a few background questions about yourself.** *Explain the purpose of the demographic information*  Can you kindly share your Profession, Years of professional experience, and organizational affiliation?  **If we were to quote you in a publication, how can we describe you**? Examples might include Viral hepatitis expert, Government employee, or respondent. **You can also choose to not be quoted at all.** |  |

1. **Organization background and testing context**

| 2.1 | | **Can you begin by briefly telling us about you and your organization’s role within viral hepatitis elimination?** | How long have you been in this role?  What is your current role within the organization?  *[If applicable]* What services does your organization provide?  Do you provide testing to the general public or specific groups at risk? Why?  How do you engage with these groups or key populations for hepatitis testing? | |
| --- | --- | --- | --- | --- |
| **Thank you for that introduction. We want to focus on testing for viral hepatitis as a key component for viral hepatitis elimination. Particularly, we are interested in any changes over the last year, while COVID-19 has impacted countries around the world.** | | | | |
| 2.2 | **Can you tell us if COVID-19 has had any impact on viral hepatitis testing in Uzbekistan? How?** | | | Can you describe in more detail how the general viral hepatitis situation in Uzbekistan has changed over the last three-years?  Has this had any implications for viral hepatitis testing in Uzbekistan?  Are there areas of viral hepatitis testing that you believe have been more impacted by COVID-19 than others?  Has your organization’s work in terms of viral hepatitis testing changed? If so, how?  *Probe: work with other organizations* |

1. **Challenges for viral hepatitis testing - Barriers**

| **We are particularly interested in any challenges that exist for viral hepatitis testing.** | | |
| --- | --- | --- |
| 3.1 | **From your point of view, what do you think are the biggest obstacles to viral hepatitis testing in Uzbekistan? This can be anything from specific services or structures to organizations or policies.** | Which aspect of your organization’s work on viral hepatitis testing is difficult?  What makes your organization’s work on viral hepatitis testing difficult?  Do healthcare facilities face challenges to provide testing? If so, which kind of challenges?  *Probe: organizational; supplies / stocks; logistic; training; personnel-related; general attitudes*  Are there other challenges people face to receive viral hepatitis testing? |
| 3.2 | **Can you describe any differences in the challenges to achieving high rates of viral hepatitis testing in the general population or in specific population groups?** | What challenges does your organization face to reach certain groups?  Do you find it challenging to motivate people to come for hepatitis testing? |

1. **Viral hepatitis Testing Successes and Opportunities - Facilitators**

| **We have talked about the challenges of viral hepatitis testing. We would now like to hear what is supportive of viral hepatitis testing in Uzbekistan.** | | |
| --- | --- | --- |
| 4.1 | **What do you think is supportive of viral hepatitis testing in Uzbekistan?** | What or who supports your organization’s work on viral hepatitis testing?  What helps healthcare facilities to provide viral hepatitis testing?  *Probe: organizational; supplies; logistic; personnel-related*  Is there anything else that might help individuals to receive viral hepatitis testing?  What encourages people to go for viral hepatitis testing? |
| 4.2 | **Have you noticed any changes to viral hepatitis testing over the last three years that were positive? What kind?** | What do you believe was conducive for these changes to take place?  What do you think led to these changes?  Who led these changes and put them in place? |
| 4.3 | **How would you assess the political commitment to increasing viral hepatitis testing in Uzbekistan?** | *If political commitment is assessed to be limited:* What might be the reason that political commitment to viral hepatitis testing is low?  Are there specific policies, guidelines, laws or processes that need to be changed? Which ones? |

1. **Improving viral hepatitis testing in Uzbekistan**

| 5.1 | **How do you think viral hepatitis testing in Uzbekistan could be improved?**  **How do you see the future for viral hepatitis testing in Uzbekistan?** | What do you think can be learned from the COVID-19 pandemic to improve and ensure viral hepatitis testing in Uzbekistan?  If you had to decide on one key thing that should be done to improve viral hepatitis testing in Uzbekistan, which one would you chose? |
| --- | --- | --- |

1. **Closing**

| 6.1 | **Is there anything else you would to share with us that we have not discussed but you think is important?** |  |
| --- | --- | --- |
| 6.2 | **Before we end I would like to give you the opportunity to ask any questions about the study.** |  |
| - Thank the key informant! - Reminder of contact details and option to get in touch if any issues come up in retrospect | | |
